# Bacteriophage Power: Next-Gen Biocontrol Strategies for Safer Meat

**DOI:** 10.3390/molecules30173641

**Published:** 2025-09-06

**Authors:** Magdalena Efenberger-Szmechtyk, Agnieszka Nowak

**Affiliations:** Institute of Fermentation Technology and Microbiology, Lodz University of Technology, Wolczanska 171/173, 90-530 Lodz, Poland

**Keywords:** bacteriophages, meat, pathogenic bacteria, synergistic effect, endolysins, spoilage bacteria

## Abstract

Lytic bacteriophages, viruses that attack and kill bacteria cells, can be used in food as biocontrol agents to prevent the growth of pathogenic bacteria. Meat is highly susceptible to bacterial growth, including pathogenic species, the control of which is crucial. Antibiotic use by breeders has resulted in bacterial resistance, which remains a huge problem; bacteriophages have emerged as an interesting alternative. In the literature, the influence of bacteriophages on common foodborne pathogens, such as *Salmonella* sp., *Listeria monocytogenes*, *Campylobacter jejuni*, *Yersinia enterocolitica*, *Escherichia coli*, and *Shigella* sp., has been described. Some phage preparations can show synergistic effects when used with other antimicrobial agents. However, data on the use of bacteriophages to inhibit the growth of meat spoilage bacteria are limited. Bacteriophages can also synthesize endolysins, which possess antimicrobial properties. Contrary to bacteriophages, which are active against only a narrow range of microorganisms (usually one bacterial species), endolysins show a broad spectrum of activity.

## 1. Introduction

Foodborne diseases have become a major global problem in recent years. According to EFSA, in 2023, the dominant zoonosis was campylobacteriosis, which constituted 58.9% of all reported cases. The second leading zoonosis was salmonellosis, followed by Shiga toxin-producing (STEC) *Escherichia coli* infections, yersiniosis, and listeriosis. Meat, especially poultry, is the main reservoir of pathogenic bacteria and the most common cause of food poisoning [1].

Animal infections are inevitable and contribute to huge economic losses for breeders. Therefore, breeders commonly use antibiotics as feed additives, which, unfortunately, contribute to the increase in bacterial resistance. The growing number of multidrug-resistant (MDR) bacteria is now becoming a serious problem. Currently, there is great interest in searching for new methods for the biocontrol of pathogens to limit antibiotic use in animal farms [2,3]. Bacteriophages have emerged as a promising solution.

Bacteriophages are viruses that attack bacteria. The phage specificity—the ability to bind to target bacteria—depends on host cell surface receptors, such as flagella, capsules, slime layers, lipopolysaccharides, and outer membrane proteins. Both lytic and lysogenic strains of bacteriophages are known. In the lysogenic cycle, the bacteriophage genome replicates without destroying the host cell. However, in the food industry, only lytic phages are recommended for use. After infection, lytic bacteriophages cause the lysis of the host cell. The lytic cycle includes (1) adsorption of the phage molecule to the host cell surface, (2) injection of phage DNA into the cell interior, (3) phage genome cyclization, (4) early phage gene expression, which leads to the control of bacterial metabolism, (5) phage genome replication, along with degradation of the bacterial genome, (6) expression of late genes for the production of proteins (endolysins), (7) maturation of bacteriophages, (8) lysis of the bacterial cell and release of descendant bacteriophages [4].

Bacteriophages are now successfully used in medicine, especially for treating infections caused by MDR microorganisms, e.g., *Staphylococcus aureus*, *Pseudomonas aeruginosa*, and *Klebsiella pneumoniae* strain*s* [5]. Interestingly, bacteriophages have also been applied as biocontrol agents in meat. Studies show that bacteriophages can inhibit the growth of foodborne pathogens, including *Salmonella* sp., *Campylobacter jejuni*, *Escherichia coli* O157:H7, *Listeria monocytogenes*, *Shigella* sp., and *Yersinia enterocolitica.* Consequently, they can increase food safety and prevent foodborne infections [6,7,8,9,10].

**Figure 1:**
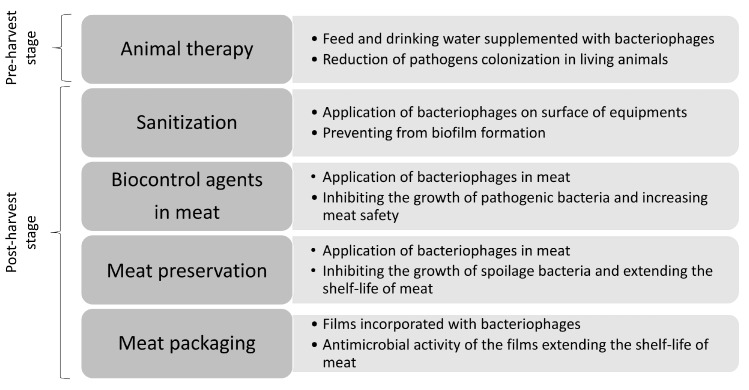
The application of bacteriophages in meat industry [11,12,13].

In the meat industry, bacteriophages can be used as protective agents at various stages of the food chain (Figure 1). First, they can be applied during primary production to eliminate diseases in animals by adding them to the feed or drinking water [11]. Second, they can be applied at the post-harvest stage as disinfectants by spraying them on the surface of equipment, preventing biofilm formation. They can also be added to meat to inhibit the growth of pathogenic and spoilage bacteria [12]. Finally, bacteriophages have been applied in active films to suppress spoilage processes of meat [13].

**Figure 2:**
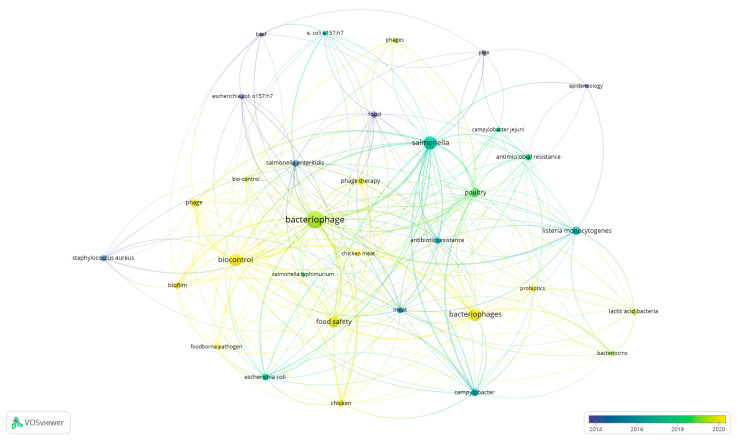
Interrelationship analysis performed with VOS viewer 1.6.20 based on review literature regarding “bacteriophages” and “meat”.

The preparation of this article began with a literature review. A dataset was prepared by searching the Scopus database using the keywords “bacteriophage” and “meat,” with no temporal constraints (Figure 2). As a result, 769 documents were identified, including 611 original articles, 104 review articles, and 32 book chapters. The remainder were conference proceedings, editorial notes, and short research descriptions. Most of the items were published in English (728). The first publication on this topic was in 1959, and its popularity has grown significantly since 2019. The final element of the bibliometric analysis was the analysis of keyword co-occurrence. Results selected from the database were entered into VOS viewer 1.6.20 for interrelationship analysis. The goal was to identify key research areas, thematic trends, and interrelationships between concepts found in scientific literature. The analysis was conducted based on the authors’ keywords. A total of 1446 original keywords were identified, 33 of which appeared at least 10 times. The five most frequently occurring words were bacteriophage, *Salmonella* sp., poultry, biocontrol, and food safety. To develop a correlation map, the analysis was narrowed to words that appeared at least five times. The results of the analysis are presented in Figure 2. Five research clusters have been identified: (1) bacteriophages in the biocontrol of chicken meat pathogens, (2) public health and resistance, (3) phage therapy and food safety, (4) natural mechanisms for safeguarding meat from contamination, and (5) innovative ways of decontaminating red meat. Cluster analysis reveals that bacteriophages have a wide range of applications in the meat industry, including biocontrol and food safety. Applications for removing infections in poultry and red meat, as well as combating antibiotic resistance, are especially essential. Many studies highlight the synergistic impact of bacteriophages on probiotics and bacteriocins. A chronological analysis shows that research has progressed from traditional studies on specific pathogens (e.g., *Salmonella* sp., *Listeria* sp., *Staphylococcus* sp.) and general bacteriophage characteristics to the practical application of phages in biocontrol, food safety, and phage therapy—particularly in the context of rising antibiotic resistance. A clear research gap was identified regarding the use of bacteriophages to control the growth of spoilage microorganisms in meat.

Based on bibliometric analysis and identified research gaps, this review explores the practical application of bacteriophages and endolysins as biocontrol agents in meat and meat products. It examines the basic requirements that bacteriophages must meet for their use in food systems, with a particular focus on regulatory frameworks in the European Union. The article also presents future perspectives in this field. Particular emphasis is placed on assessing the impact of bacteriophages and endolysins on the growth of pathogenic and spoilage bacteria, highlighting their potential to improve food safety and extend shelf life. Detailed literature data about application of bacteriophages in meat are presented in Table S1.

## 2. Requirements for Bacteriophages Used in Food

**Figure 3:**
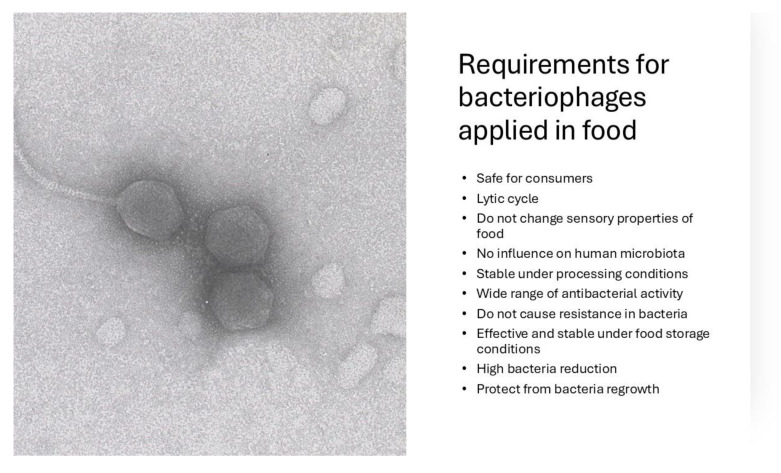
Requirements for bacteriophages applied in food [7,14].

Strict requirements for bacteriophages intended for use in food have not yet been established. However, according to literature data and studies concerning the application of bacteriophages in food, some conditions have been proposed (Figure 3).

First, only lytic viruses that contribute to host cell death are permitted to be used in food. Bacteriophages must be entirely safe for consumers and not pose a risk to human health. Genotoxic and cytotoxic studies must be performed on eukaryotic cells. Generally, toxicity studies have not shown any toxic effects, and phages do not have any unfavorable effects on human health. Phage preparations should also not cause any respiratory sensitization. Although phage preparations may contain bacterial endotoxins, their levels are expected to be very low and not pose a risk via inhalation [7,14].

Bacteriophages should be highly specific and contrary to antibiotics, must not influence the human commensal microbiota. Most bacteriophages are monovalent and active against only a single bacterial species, strain, or serovar [15]. Bacteriophages generally do not kill beneficial bacteria, including the human commensal microbiota, probiotic bacteria, and microorganisms added during technological processes. Thus, they can also be successfully used in fermented products.

On the other hand, bacteriophages should exert broad lytic activity and be effective against many bacterial strains. Due to their narrow host range, phage preparations are often a mixture of bacteriophages. A phage cocktail has stronger lytic properties than individual phages [16,17]. However, some studies have shown that bacteriophages can be active against different bacterial species. For instance, a phage specific for *Salmonella enterica* was also capable of lysing *Escherichia coli* 0157:H7 [18]. The commercial phage preparation PhageGuard L^TM^ (Phageguard Micreos Food Safety, Wageningen, The Netherlands) targeting pathogenic *Listeria monocytogenes*, was also active against non-pathogenic *Listeria innocua* [19].

Bacteriophages should reduce the counts of target microorganisms to a relatively high degree. PhageGuard L^TM^ is supposed to reduce *L. monocytogenes* number by >1 log units in ready-to-eat meat products. PhageGuard S^TM^ (Phageguard Micreos Food Safety, Wageningen, The Netherlands) can reduce the number of *Salmonella* sp. by up to 2 log units in fresh poultry meats and beef. According to the literature, bacteriophages usually decrease the host bacteria number by about 1–2 log units, although in some cases, a reduction of about 6 log units was observed [20,21,22].

Stability over a broad pH and temperature range is also a very important factor, as it determines whether bacteriophages are stable in different types of food and whether they are sensitive to pH changes during food storage or different food processing conditions. Generally, studies show that phages are stable at pH 4–11 and temperatures up to 65 °C [17,23,24]. This is important because this pH range is typical for most food products. Thermal stability is important for heat-treated products. However, Karaynir et al. [25] reported that some phages can exhibit relatively low stability. Phage titer was observed to decrease by about 2 log units at pH 6–10. At pH 4 and 12, complete inactivation of bacteriophages was detected. Phages were also heat-unstable and lost their activity after 5 min of incubation at 60 °C. These data show that investigating bacteriophages’ pH and temperature stability is of great importance.

Phages should also be effective and stable under food storage conditions. SalmoFresh^TM^ (Intralytix, Columbia, MD, USA) is expected to be effective at 2–42 °C [26], and PhageGuard S^TM^ at 0–35 °C [27]. This suggests that phage preparations show lytic activity in food stored both at room temperature and when refrigerated.

The literature data report that some bacteria are unfortunately resistant to bacteriophages. Scientists claim that the emergence of phage-resistant bacteria is inevitable. However, selective pressure on bacteriophages can overcome bacterial mutations responsible for resistance mechanisms [28,29].

Studies show that bacteriophages can exert synergistic effects when used in combination with antimicrobial substances. However, some antimicrobials have antiviral properties and reduce phage numbers. Therefore, phages should be stable in the presence of antimicrobial agents. Only minor reductions in phage numbers can be accepted [30].

## 3. Law Barriers in European Union

Although foodborne diseases are a serious global problem, the number of multidrug-resistant bacteria is still growing, and there is a great need to develop new methods for eliminating pathogenic bacteria. The use of bacteriophages in any category of food is not yet allowed in the European Union. According to Regulation (EC) No 1333/2008 of the European Parliament and of the Council of 16 December 2008 on food additives, bacteriophages are not included in the list of additives allowed for use in food as biopreservation agents. Endolysins are not permitted either [31].

In 2009 and 2017, EFSA excluded bacteriophages from QPS (qualified presumption of safety) assessment. The justification was the lack of a single universal classification system, and phages are not precisely allocated to genera or species. In addition, a distinction between temperate and virulent phages and the possible carriage of undesirable genes in their genomes still need further study. Possible recombination between phages might allow gene-shuffling, which might lead to subsequent changes in host ranges and their virulent/lysogenic lifestyle. In addition, bacteriophages may function as carriers of genetic material. Phage infection may integrate antimicrobial resistance genes and virulence factors into bacterial genomes, which can cause bacterial resistance to antibiotics or the production of disease-causing toxins. Moreover, many genes encoding proteins do not have counterparts in the databases [32,33]. In 2023, EFSA supported the previous decision, and bacteriophages remain excluded from QPS assessment. It was emphasized that although phages have already been implemented in food and feed chains, mainly outside Europe, and no consumer health problems have emerged, too many concerns remain to allow their application [34].

However, in some countries, such as the USA, Brazil, The Netherlands, Israel, Canada, Switzerland, Australia, and New Zealand, phage preparations have been approved as auxiliary agents in the control of pathogens in food. Some phage preparations are already available on the market (Table 1). Companies such as Phageguard Micreos Food Safety, Intralytix, and Phagelux offer commercial phage preparations aimed at suppressing the growth of *Salmonella* sp., *Listeria monocytogenes*, *Escherichia coli*, and *Shigella* sp. They are intended for use in meat, ready-to-eat meat products, fish, eggs, and dairy products, as well as fruits and vegetables, including processed products. Some of them possess GRAS (Generally Recognized as Safe) status, have Kosher and HALAL certification, and can be used in organic food. They have been approved by the FDA (Food and Drug Administration) and USDA (United States Department of Agriculture) [35]. The only phage preparation that is close to being approved by EFSA is PhageGuard L^TM^. In 2016, EFSA gave the scientific opinion that Phage Guard L^TM^ P100 is safe and does not pose a risk to human health. However, more studies on its efficacy are recommended. EFSA emphasized that bacterial reduction in ready-to-eat food is uncertain and not sufficient, and their regrowth may occur after treatment. The mean bacteria reduction ranged from 1.7 to 3.4 log CFU/g at the maximum dose. In addition, it was claimed that some *Listeria* strains (2.5–9.5%) are resistant to phage P100. Therefore, Phage Guard L^TM^ should only be considered as an additional tool to further reduce the load of *L. monocytogenes* while following GHP and GMP, not as a substitute for those practices. Therefore, currently, more studies must be performed before approval [36].

Bacteriophage use was also tested in the pre-harvest stage. Phages can be added to animal feed, protecting animals from infections and consequently ensuring meat safety. Regulation (EC) No 1831/2003 of the European Parliament and of the Council of 22 September 2003 on additives for use in animal nutrition states that all feed additives must be included in the European Union Register of Feed Additives [37]. Bacteriophages and endolysins are not listed in the EU Register and therefore cannot be applied to animal feed. Nevertheless, the phage preparation Bafasal^®^ produced by the Polish company Proteon Pharmaceuticals received a positive EFSA opinion. Bafasal^®^ contains four lytic bacteriophages, PCM F/00069, PCM F/00070, PCM F/00071, and PCM F/00097, infecting *Salmonella* Gallinarum B/00111. The preparation is intended for all avian species and can be used as an additive in drinking water and liquid complementary feed. EFSA claimed that Bafasal^®^ is safe for all avian species and the environment, and it is not expected to pose a risk to consumers. Bafasal^®^ is not a skin or eye irritant but should be considered a potential skin and respiratory sensitizer, and inhalation and dermal exposure are considered risks. Based on the new data provided, the Panel concluded that Bafasal^®^ has the potential to reduce *S.* Enteritidis when used in feed and water for all poultry species [38,39,40]. The product is already registered in India and Brazil, with regulatory approval processes ongoing in several other countries. On 15 July 2025, Bafasal received full regulatory approval from the European Union for use in animal nutrition [41]. Notably, it is the first phage preparation approved for use in the EU. This is extremely important for both the poultry industry and scientific research on phages.

## 4. Application of Bacteriophages to Control the Growth of Pathogenic Bacteria in Meat

Pathogenic bacteria pose a serious risk to food safety and contribute to economic losses for manufacturers. Meat is the main source of food poisoning, and therefore, new methods suppressing the growth of pathogenic bacteria are intensively studied. The application of bacteriophages as biocontrol agents against pathogenic bacteria in meat is currently of great interest; however, more studies are needed to fully leverage the lytic potential of bacteriophages.

According to the available literature data, although studies conducted in broth show strong antibacterial activity of bacteriophages, the lytic activity of bacteriophages in a solid meat matrix is usually weaker than in liquid broth. This is probably due to the difficulty bacteriophages have accessing host bacteria and the presence of certain meat compounds, which can have protective effects on microbial cells [16]. The meat storage temperature in experiments also plays an important role. The reduction in bacterial numbers at refrigeration temperature is lower than at higher temperatures. This is due to the slower growth of microorganisms, not necessarily weaker lytic activity. However, it has also been suggested that bacteriophages do not replicate at low temperatures [16]. Studies show that endolysins—toxins produced in the phage lytic cycle—remain active at 4 °C [42]. The literature data show that the effect of bacteriophages on bacterial counts depends on the contamination level and the phage concentration in the stock suspension. MOI (multiplicity of infection) is a parameter describing the average number of bacteriophages per bacterium. Studies generally show that a higher MOI increases the likelihood of bacteriophage–bacteria contact and enhances lytic activity [16,17,43].

### 4.1. Salmonella sp.

*Salmonella* sp. is detected mainly in meat and meat products, milk and milk products, fruit and vegetables, fish and fishery products, and eggs and egg products. It is the second leading cause of zoonosis. The most infected animals are chickens, followed by cattle, turkeys, pigs, ducks, and geese. The most prevalent infections are caused by *Salmonella enterica* Enteritidis and *Salmonella enterica* Typhimurium [1].

*Salmonella* sp. is a common problem, especially in poultry farms and the poultry industry. The methods of eliminating *Salmonella* sp., such as chemical disinfectants, heat processing, and UV treatment, although effective, are not entirely safe, and they negatively affect food quality. Bacteriophages seem to be an interesting alternative to control *Salmonella* sp. growth.

In a recent study, Kumar et al. [43] showed the effect of bacteriophages on *S.* Typhimurium growth in chicken meat. At 25 °C, the phage cocktail diminished the bacterial number by 1.2–1.3 log units after 7 h of storage, depending on the phage titer. At 4 °C, the most significant reduction of 1.3–2.5 log units was achieved on day 7, although the influence was detected as early as 24 h. Aguilera et al. [44] reported that the number of *S.* Typhimurium in chicken meat decreased by about 1 log unit after 24 h of storage at 22 and 30 °C. At 10 °C, a reduction of 1.2–1.5 log units was achieved during 72 h of storage. According to Thung et al. [23], during 24 h of storage at 4 °C, the population of *S.* Enteritidis in beef and chicken meat was reduced by 2.1 and 2.0 log units, respectively, compared to the initial population. In addition, when compared to the control, the bacterial loads in phage-treated samples were 1.8–2.2 log units lower.

Demirarslan et al. [22] investigated the effect of phages at a ~10^9^ PFU/g dose on chicken breast and skin contaminated with *Salmonella* sp. (~10^3^ and ~10^6^ CFU/g) during chilled storage (4 °C for 8 days) and at room temperature (25 °C for 4 days). Bacteriophages alone and in a cocktail were effective in preventing *Salmonella* sp. growth when both low and high bacterial inoculation doses were applied. In samples containing *Salmonella* sp. at 10^3^ CFU/g, phages decreased bacterial counts to undetectable levels during the storage period independently of temperature. In samples inoculated with *Salmonella* sp. at 10^6^ CFU/g, a reduction of 4.1–6.4 log units on the first day of storage was observed. Furthermore, bacteriophages also prevented subsequent *Salmonella* sp. contamination at 4 °C after inoculation with both high and low *Salmonella* sp. doses. The study results presented by Demirarslan et al. [22] are very promising and show strong antibacterial activity of bacteriophages, even when the inoculum contains a high bacterial count. This is very important because most studies report reductions of about 1–2 log units. It should also be emphasized that the authors did not observe *Salmonella* sp. regrowth.

Studies show that bacteriophages can also be applied in processed and fermented meat products. Galarce et al. [45] applied a bacteriophage cocktail composed of five lytic phages to reduce the number of *S.* Enteritidis in the following processed meat products: cooked (turkey ham and chicken sausage) and cured sausage (Italian salami and barbecue sausage). The products were incubated for 10 days at 4 and 18 °C. Bacteriophages statistically significantly reduced *S.* Enteritidis counts, depending on the food matrix. The greatest reduction was detected in barbecued sausage (reduced by 1.5–2.1 log units), whereas at 4 °C, the highest decrease was observed in turkey ham (reduced by 1.6–2.1 log units). These findings suggest that phage titers decreased during storage, which is an undesirable effect. Food matrix components could inactivate or immobilize bacteriophages in food with low water activity, or their diffusion could be insufficient. However, other studies showed that bacteriophage levels can remain stable during storage independently of the food matrix or storage temperature [46,47,48]. Notably, Galarce et al. [45] analyzed processed meat products and found that the processing conditions could also influence the number of bacteriophages and their lytic properties.

There are some commercial phage preparations against *Salmonella* sp. intended for use in food, including meat, such as SalmoFresh^TM^ and PhageGuard S^TM^. According to Sharma et al. [49], the bacteriophage preparation SalmoFresh^TM^ was the most effective against *S.* Typhimurium among the tested serotypes (Kentucky Enteritidis and Heidelberg) at 37 °C in TSB medium. The phage solution reduced viable counts of *S.* Typhimurium at the lower MOI studied (1, 10^2^, and 10^3^ PFU/CFU), whereas other serovars were inhibited using the highest MOI (10^4^ PFU/CFU). Sukumaran et al. [50] investigated the effect of the phage preparation SalmoFresh^TM^ on *Salmonella* sp. inoculated on chicken breast meat. Surface and dip applications of bacteriophages were studied. After the dip application, the phage cocktail reduced bacterial counts by 0.7 and 0.9 on days 0 and 1 of storage compared to the control. After surface treatment, SalmoFresh^TM^ reduced bacterial counts by 0.8–1.0 log units during 7 days of storage under aerobic conditions and by 1.1–1.2 when stored in MAP compared to the control. The effectiveness of SalmoFresh^TM^ was also studied by Sharma et al. [49]. The phage preparation was spread on turkey breast cutlets and on ground turkey. The meat was inoculated with *S.* Heidenberg. A reduction of 0.6–1.3 log units (compared to the control) during 7 days of storage was observed. However, the phage cocktail did not show a significant effect on ground turkey before grinding. Grant et al. [51] compared the sensitivity of *Salmonella* sp. isolated and non-isolated from chicken meat. They indicated that the isolates were more resistant to the phage preparation PhageGuard S™. After 30 min and 8 h, the non-isolates were reduced by 0.7 log and 0.9 log units, respectively, and isolates decreased by 0.4 and 0.7 log units. Kim, J.H. et al. [17] revealed that a phage cocktail at MOI = 10^4^, 10^5^, or 10^6^ PFU/CFU decreased the counts of *S.* Enteritidis in chicken breast meat during storage at 4 °C. As expected, the greatest influence was achieved using the highest MOI. At MOI = 10^6^ PFU/CFU, bacteriophages reduced the bacterial load by 1.3–2.9 log units during 7 days of storage.

Some studies compared the effect of bacteriophages with antimicrobial substances used in the meat industry. Wang et al. [52] reported that bacteriophages exert a stronger inhibitory effect on *S.* Typhimurium than potassium sorbate—a commonly used chemical preservative. This suggests that phages can be an alternative to chemical preservatives. Studies showed that phages reduced *S.* Typhimurium numbers in ready-to–eat (RTE) duck meat by 0.8–5.8 log units compared to the control, whereas potassium sorbate treatment resulted in a minor reduction of 0.3–1 log units. At MOI = 10^6^ (4 and 25 °C) and 10^5^ (4 °C), bacteriophages completely inactivated *S.* Typhimurium for the whole storage period. Aykın-Dinçer et al. [53] compared the effects of lactic acid—also approved as a food preservative—bacteriophages, and ultrasound on turkey breast slices kept in aerobic and vacuum conditions. After 6 h, bacteriophages reduced *S. enterica* counts by 1.3 (aerobic atmosphere) and 1.4 (vacuum atmosphere) log units. Lactic acid had only a slightly stronger effect, and ultrasound resulted in the least inhibition. Sukumaran et al. [50] demonstrated that the phage preparation SalmoFresh^TM^ was slightly more effective in reducing *Salmonella* sp. numbers than other chemical antimicrobials, such as peracetic acid and cetylpyridinium chloride. Shebs-Maurine et al. [54] compared the effectiveness of peroxyacetic acid and the commercial phage preparation PhageGuard S^TM^ against *Salmonella* sp. on ground beef when applied directly on trimmings and at different grinding stages. Peroxyacetic acid did not affect bacterial counts in any variants. However, the phage cocktail at both concentrations (10^8^ and 10^9^ PFU/g) significantly reduced *Salmonella* sp. counts by 0.8–1.7 log units. Yeh et al. [55] showed that PhageGuard S^TM^ reduced bacterial counts by about 1 log unit in ground beef, pork, chicken, and turkey.

The recent study by Khan et al. [56] showed that the application of bacteriophages decreased *S.* Typhimurium numbers by 1.2, 2.2, and 1.5 log CFU/piece after 2, 4, and 6 h of incubation, respectively, in chicken breast samples stored at 25 °C.

As mentioned before, bacteriophages generally have a narrow host range and show lytic activity against a single bacterial species or even a single strain. However, one of the recent studies indicated that phages specific for *S. enterica* also inhibited the growth of E. coli O157:H7 in duck meat. *S. enterica* was reduced by 1.7–1.8 log units and *E. coli* by 2 log units compared to the control [18]. In addition, Hou et al. [57] demonstrated that bacteriophages specific for *S. enterica* were also active against *E. coli* O157:H7 in ready-to-eat chicken claw and pork loin stored at 4 °C for 10 h. The phages decreased both bacteria to undetectable levels (reduced by more than 4 log units) after 2 or 4 h of incubation.

### 4.2. Campylobacter sp.

Campylobacteriosis, considered to be the most prevalent zoonosis in the European Union, is caused by two *Campylobacter* species: *Campylobacter jejuni* and *Campylobacter coli*. However, the most severe infections are caused by *C. jejuni*. The most prevalent sources of *Campylobacte*r sp. infections are meat and meat products, followed by milk and milk products. *Campylobacter* spp. are part of the normal intestinal flora of many animals, mainly chickens, so poultry meat can become contaminated through feces. Uncooked chicken meat, hand-to-mouth transfer in the kitchen, and cross-contamination from other foods can lead to infections in humans. Biocontrol of *Campylobacter* sp. in food products is thus of great importance [1,58,59].

Although campylobacteriosis is a worldwide problem, the literature data on the application of bacteriophages to control *Campylobacter* sp. counts are limited. Generally, *Campylobacter* spp. are thermophilic bacteria, and their growth is inhibited at 4 °C. However, meat contaminated at earlier stages of production with *Campylobacter* spp., although refrigerated during storage, remains dangerous.

Bacteriophages targeting *Campylobacter* spp. are categorized into three groups based on their genome sizes (group I ∼320–425 kb, group II ∼184 kb, and group III ∼138 kb). Phages belonging to group II are flagellotropic, which means that they require a functional flagellum to infect cells. Group III phages are dependent on capsular polysaccharide structures. There is little knowledge about phages belonging to group I. They have rarely been isolated, and their genomes have not been sequenced yet [60]. In addition, Zampara et al. [61] reported that the most potent bacteriophages belong to group III.

Thung et al. [24] investigated the use of bacteriophages in mutton and chicken meat stored at 4 °C. Even though the MOI used in this study, 10^2^ PFU/CFU, was quite low, bacteriophages were effective in reducing bacterial numbers. After 6 h, *C. jejuni* loads in mutton and chicken meat had diminished by 0.6 and 0.5 log units, respectively, compared to the control. After 48 h, reductions of 1.5 and 1.4 log units were observed. As previously mentioned, the bacteriophage biocontrol mechanism is influenced by slower bacterial growth under refrigeration conditions; however, endolysins remain active at refrigerated conditions and participate in the phage lytic cycle.

According to Zampara et al. [62], bacteriophages reduced *C. jejuni* counts on chicken skin, although the effect was not very significant. The phage cocktail had the most potent influence and decreased bacterial numbers by only 0.7 log units after 24 h of incubation compared to the control. Orquera et al. [63] revealed that bacteriophages reduced *Campylobacter* sp. cell numbers by 1 log unit at 37° C, but only in broth. However, at 4 °C, no influence was observed in either broth or meat. Firlieyanti et al. [64] applied bacteriophages to chicken liver homogenates stored at 4 °C, and the effect was small, although statistically significant. When a high bacterial count of 5 log CFU/g was used in the inoculum, the reduction in *Campylobacter* sp. counts was 0.2–0.7 log units.

The literature data generally reveal that the influence of phages on *Campylobacter* spp. in meat is quite weak [62,63,64], especially when we compare the results obtained for *Salmonella* sp. In meat, the reduction in *Campylobacter* counts does not usually exceed 1 log unit, whereas *Salmonella* sp. numbers were reduced by more than 1 log unit [16,17,22,23,48], even exceeding 6 log units [22]. Only in the most recent study by Xiao et al. [65] did bacteriophages reduce the number of *C. jejuni* on chicken skin to undetectable levels, which were observed after 24 h and 48 h of incubation at 4 °C. The results are promising, suggesting that searching for bacteriophages with strong lytic activity against *Campylobacter* spp. is warranted.

Some literature data demonstrate the pre-harvest treatment of animals with phages, which appears to be a promising research direction.

In broiler farms, *Campylobacter* sp. infections are a serious problem. Broiler flocks become infected very quickly due to the rapid spread of *Campylobacter* spp. In addition, broiler flocks usually comprise more than 10,000 birds, which makes campylobacteriosis very problematic for breeders. Biosecurity measures should be taken to reduce the introduction of *Campylobacter* spp. via animals, farmers, and visitors [66]. According to Georgiev, Beauvais, and Guitian [67], biosecurity measures decreased campylobacteriosis in flocks by 53–86%. Smith et al. [68] reported that in farms with high hygiene standards, the *Campylobacter* sp. prevalence was 20–40% lower compared to farms with lower standards.

Kittler et al. [69] applied a phage cocktail in drinking water (log 5.8 to 7.5 PFU/bird). After 1 day, *Campylobacter* sp. counts in fecal samples were reduced to below the detection limit (<50 CFU/g). At slaughter, a significant reduction (>3.2 log units) in cecal samples compared to the control group was still observed. In addition, the results showed that the maximum reduction was achieved when bacteriophages were applied 2–4 days prior to slaughter. D’Angelantonio et al. [70] demonstrated the effect of bacteriophages added to feed on *C. jejuni* levels in broiler chickens. On day 40, significant differences between control and experimental groups treated with phages at MOI = 10^7^ and 10^8^ PFU/mL were detected. In the lower intestine of control birds, the *C. jejuni* number was 10^8^ CFU/g of cecal content. In the experimental groups, the *C. jejuni* count was 10^7^ CFU/g of cecal content (MOI = 10^7^ PFU/mL) and 10^6^ CFU/g of cecal content (MOI = 10^8^ PFU/mL). Chinivasagam et al. [71] showed that *Campylobacter* sp. levels in the ceca of broilers treated with phages decreased by 1–3 log units compared to controls. Nevertheless, some individual birds showed quite high cecal *Campylobacter* sp. levels and low phage titers, suggesting that a longer period of phage treatment (>24 h) may be required to ensure phage replication and effective biocontrol.

### 4.3. Listeria monocytogenes

*L. monocytogenes* is not a predominant foodborne pathogen; however, listeriosis is a serious danger with a high mortality rate, especially for at-risk individuals such as pregnant women, neonates, immunocompromised people, and the elderly. The number of listeriosis cases increased significantly in 2019, with a 50% increase compared to 2018. The number of fatalities caused by *L. monocytogenes* infections was also higher in 2019. Listeriosis is therefore considered by EFSA to be the most severe foodborne disease. Unpasteurized food products, including milk and meat products, as well as raw fish and seafood products, are reported to support the growth of *L. monocytogenes.* Among meats, the main source of listeriosis is pork meat. However, bovines, broilers, and turkeys can also be infected [1]. Due to the severity of the illnesses and the growing number of fatalities caused by *Listeria monocytogenes*, controlling its growth in food is crucial.

Some commercial phage preparations, such as Phage Guard L^TM^ and ListShield^TM^, have been approved for use as biocontrol agents of *L. monocytogenes* growth in meat. Ishaq et al. [21] investigated the effect of the ListShield^TM^ phage preparation on the surface of raw beef meat stored at 4 °C. In the control inoculated with *L. monocytogenes*, the bacterial number increased during storage, reaching 8.7 log CFU/g on day 15. Nevertheless, in the samples treated with bacteriophages, *L. monocytogene*s counts decreased from 5.2 to 2.9 log CFU/g during storage. Moreover, the phages did not negatively affect the color or pH value of beef meat. The bacteriophage preparation also decreased purge loss, possibly because reducing the bacterial activity maintained the muscle integrity and improved the water-holding capacity. Lower nitrogenous losses during storage were also detected. Nitrogenous losses were probably due to increased microbial activity, which resulted in protein degradation and nitrogenous losses. The results suggest that bacteriophages improved the microbial quality of meat and the physicochemical parameters of meat stability.

Gutiérrez et al. [72] compared the effectiveness of the commercial phage preparations ListShield^TM^ and Phage Guard L^TM^ in Spanish dry-cured ham. ListShield™ caused the lysis of 100% of *L. monocytogenes* strains tested, whereas Phage Guard L™ lysed only 64% of strains. However, it should be noted that the phage titer of Phage Guard L™ recommended for use was lower than that of ListShield™. In dry-cured ham, Phage Guard L™ completely inactivated bacteria after 24 h of incubation at 4 and 12 °C, regardless of the inoculum size. ListShield™ also completely inhibited the growth of *L. monocytogenes*, except for in samples contaminated with a high bacterial load (10^5^ CFU/cm^2^). At 4 °C, a decrease of 3.5 log units was observed after 14 days of incubation, and at 12 °C, the antibacterial activity of ListShield™ was very low.

Ahmadi et al. [73] investigated the inhibitory effect of bacteriophages on *L. monocytogenes* and demonstrated that they were only effective when meat was inoculated after cooking. The meat was stored at 4 °C under vacuum conditions. After 30 min, bacterial counts were reduced by approximately 1.5 log units and continued to fall. After 24 h, the bacterial load was below the detection limit, where it remained for 7 days. Then, *L. monocytogenes* regrowth was observed, reaching a significant number on day 10. Pathogen regrowth is a huge problem and should be prevented in meat. In this study, it is likely that *L. monocytogenes* cells that had not contacted the phage began to proliferate. When treatment was applied prior to cooking, no changes in phage titer during 28 days of refrigerated storage were observed, which suggests that phages are stable after heat treatment at 65 °C. The authors suggest that a different inactivation mechanism occurred in their study. When substantial numbers of phages adsorb on the cell surface, significant cell damage occurs, resulting in lysis without the production of progeny phages. In this study, a high MOI = 10^5^ was used, which supports the theory. Liu et al. [74] demonstrated that bacteriophages effectively inhibited the growth of L. monocytogenes in beef meat depending on the phage titer. Reductions of 2.9 log CFU/sample, 2.6 log CFU/sample, and 1.5 log CFU/sample were observed after 18 h incubation with the phage lysate at MOIs of 10000, 1000, and 100, respectively.

### 4.4. Escherichia coli

Some strains of *Escherichia coli* are dangerous human pathogens. The most severe are Shiga toxin-producing *E. coli* (STEC). They cause hemorrhagic enterocolitis, hemolytic uremic syndrome (HUS), and thrombotic thrombocytopenic purpura. STEC *E. coli* produces Shiga toxins and AB-type toxins, which inhibit protein synthesis. Shiga toxins produced in the intestines can enter the systemic circulation, which can result in kidney, jejunum, and liver damage. Multidrug resistance, a wide range of adaptability features, and extremely high infectivity make STEC *E. coli* infections very dangerous [75,76]. The literature data reveal that lytic bacteriophages can be effective agents for biocontrol of STEC *E. coli* growth.

The commercial phage preparations EcoShield PX^TM^ and PhageGuard E^TM^ have been designed to control the growth of pathogenic *E. coli*. Carter et al. [46] investigated the effect of the commercial phage preparation EcoShield PX^TM^ on *E. coli* (STEC) O157:H7 in beef meat. The phage cocktail reduced bacterial levels by at least 94% after 5 min. Nevertheless, it did not prevent recontamination. Shebs et al. [77] compared the effectiveness of the phage preparation PhageGuard E^TM^ and organic acids (lactic acid and peroxyacetic acid) in vacuum-packaged beef and beef stored aerobically. The bacteriophages were more effective than organic acids, which did not show antibacterial activity in meat kept under vacuum conditions. PhageGuard E^TM^ reduced bacterial counts by 1.4 log units after 6 h in both storage atmospheres.

Some differences in the lytic activity of bacteriophages can be observed among different types of meat. This could be due to different moisture content. Lower water content can decrease the lytic ability of bacteriophages by reducing their diffusion in the food matrix and, consequently, their ability to diffuse into the host bacterial cells. Seo et al. [78] applied the bacteriophage BPECO19 in beef, pork, and chicken meat to inhibit the growth of *E. coli* O157:H7. At MOI = 10^5^ PFU/CFU, the bacteriophage completely inhibited *E. coli* O157:H7 in beef and pork meat after 4 and 8 h of exposure, respectively. In chicken meat, the bacteriophage showed slightly weaker activity and reduced viable bacterial counts by 4.7 log units after 4 h of incubation.

Bacteriophages were also used for biocontrol of ESBL (extended-spectrum beta-lactamase) *E. coli*. Minh et al. [79] investigated the influence of lytic bacteriophages in raw chicken meat contaminated with ESBL *E. coli*. In broth, a phage cocktail decreased bacterial counts by 3.4–5.1 log units during 6 h of incubation at 25 °C, and after 24 h, bacterial cells were below the detection limit (<10 CFU/mL). At 5 °C, the phage mixture reduced bacterial levels by 1.0–2.1 log units. In raw chicken meat, the phage cocktail caused a significant reduction in ESBL *E. coli* counts. At 25 °C, the bacterial load decreased by 1.7 and 2.9 log units after 2 and 24 h of exposure; at a lower temperature, reductions of 1.3 and 1.9 log units were detected, respectively. Son et al. [80] demonstrated that in raw beef contaminated with *E. coli* O157:H7, phages reduced the bacterial loads by 2.5 and 1.8 log units after 24 h of storage at 25 and 8 °C, respectively. When raw beef was contaminated with a bacterial cocktail of *E. coli* O157:H7 and ESBL *E. coli*, the effect of PE37 treatment was weaker, with decreases of 1.4 and 1.0 log units after 24 h of storage at 25 and 8 °C, respectively. At 25 °C, bacterial regrowth was observed, whereas at 8 °C, bacterial levels were stable throughout the storage period. It should be emphasized that the selected bacteriophages exerted lytic activity against 100% of *E. coli* O157:H7, 100% of STEC O8, O26, O103, and O119, and 64% of the ESBL *E. coli* (O20, O74, O153, O159, and O serogroup untypeable) strains tested.

Hudson et al. [81] compared the effect of different phage titers on *E. coli* O157:H7 in beef meat. At the lowest phage titer used, equal to 10^3^ PFU/piece of meat, no bacteria inactivation was observed. At the higher concentration of 10^5^ PFU/piece, the phages only slightly reduced the number of *E. coli*. However, at 10^7^ PFU/piece, the reduction was significant: after 1 h of exposure, the bacterial load decreased by 2.3 log units, and the host cells did not recover. Shebs-Maurine et al. [82] investigated the effect of a phage mixture applied to ground beef on the viability of a bacteria cocktail composed of all six *E. coli* STEC serotypes. The samples were analyzed after 30 min and 6 h of storage at 25 and 7 °C. The bacteriophage cocktail reduced viable counts by 0.8–1.3 and 0.8–1.0 log units compared to the control during storage for 6 h at 25 °C and at 7 °C, respectively. Witte et al. [83] reported that the phage cocktail EP75/EP335 caused the lysis of 78/88 isolates. Phages added to beef meat at concentrations of 10^7^ and 10^8^ PFU/cm^2^ reduced bacterial counts by 0.8–1.1 log units and 0.9–1.3 log units, respectively.

Tomat et al. [84] investigated the effect of a cocktail composed of six bacteriophages on DH5α, enteropathogenic (EPEC), and enterotoxigenic (ETEC) *E. coli* in beef meat. At 4 °C, a low but significant reduction (by 0.5–1.0 log units) was observed after 24 h of incubation for all bacterial strains, and after 48 h, DH5α regrowth was observed. The phage cocktail showed stronger lytic activity at higher temperatures (24 and 37 °C). At 24 °C, the bacterial load decreased by 2.6–4.0 log units, whereas at 37 °C, the reduction ranged from 3.0 to 3.8 log units after 24 h of exposure. In this experiment, phage titers were significantly reduced during storage. At 4 °C, the reduction was about 1.8 log units; at 24 °C, it ranged from 1.2 to 1.8 log units; and at 37 °C, it was about 0.6–1.4 log units. Hudson et al. [81] reported that bacteriophages do not replicate in the food matrix at low temperatures. However, at a higher temperature of 37 °C, the number of phages added to *E. coli* O157:H7 cells (37 °C) growing on beef increased after 20 h of incubation.

### 4.5. Yersinia enterocolitica

There is little literature data about the application of bacteriophages to control *Yersinia enterocolitica* growth in meat. Considering that yersiniosis is the fourth leading zoonotic disease in the EU (more common than listeriosis), there is a great need to extend the research on using bacteriophages to prevent *Y. enterocolitica* growth. Most infections are caused by consuming raw and undercooked pork meat, but also contaminated vegetables and juices, raw milk, or untreated water. The bacteria also spread through contact with other infected people, infected animals, or their feces [1,85]

The available literature data show that bacteriophages are effective in reducing *Y. enterocolitica* growth and decreasing bacterial numbers by about 2 log units. Orquera et al. [63] first demonstrated the significant influence of phages on *Y. enterocoitica* counts in meat stored at 4 °C. Bacterial numbers decreased by about 2 log units after 24 h of storage. Jun et al. [20] also reported that bacteriophages were effective in controlling the growth of *Y. enterocolitica* in pork meat. Bacterial counts decreased from 3.4 to 2.3 log CFU/g in raw pork during 72 h of storage and from 2.3 to 1.6 log CFU/g in ready-to-eat pork during 12 h of storage. In contrast, the bacterial number increased from 3 to 6 log CFU/g in both meat samples in the control. The effects were observed after 24 h and 12 h in raw pork meat and ready-to-eat pork, respectively.

### 4.6. Shigella sp.

*Shigella* sp. is also a foodborne pathogen and is classified into four serogroups: A (*Shigella dysenteriae*), B (*Shigella flexneri*), C (*Shigella boydii*), and D (*Shigella sonnei*). *S. dysenteriae* is responsible for severe shigellosis, whereas *S. sonnei* and *S. flexneri* are dominant species in outbreaks in developed and developing countries. They can be transmitted through person-to-person contact or indirectly through contaminated food and water. The sources of *Shigella* sp. infection include fresh vegetables, chicken, meat, salads, fruits, dairy products, and different water resources [86].

Soffer et al. [87] investigated the effectiveness of the commercial phage preparation ShigaShield^TM^ against *Shigella* sp. in various food products, including corned beef deli meat and pre-cooked chicken. In chicken breast strips, bacterial counts decreased by 1.6 and 0.7 log units when the phage cocktail was applied at concentrations of 9 × 10^7^ and 9 × 10^6^ PFU/g, respectively. In beef meat, reductions of 1.6 and 0.6 log units were observed, respectively. Shahin and Bouzari [88] demonstrated the application of bacteriophages as a biocontrol agent against *S. flexneri* in raw or cooked chicken breast. Phages were added after bacteria inoculation (in the treatment experiment) or before (in the prevention experiment). Bacteriophages had an influence in both cases, achieving a reduction of approximately 2 log units. Bacterial numbers decreased during the first 72 h and remained stable. In the prevention experiment, the effect was slightly stronger. Shahin et al. [89] compared the effectiveness of bacteriophages in controlling the *S. flexneri* growth in different food matrices, including cooked chicken meat. Bacterial reduction was the highest in meat. Phages caused a reduction of about 4 log units throughout the storage period (7 days).

Shahin et al. [90] also investigated the effect of bacteriophages on multidrug-resistant *S. flexneri* and *S. sonnei* in chicken meat. The bacterial number in samples treated with phages decreased after 2 h, and the largest reduction was observed after 48 h; the bacterial count continued to decline up to 120 h. The viable counts decreased from 4.2 to 1.4 and 1.1 log CFU/g when incubated with single bacteriophages. When samples were treated with the cocktail, the inhibitory effect was even stronger, and the bacterial number decreased from 4.2 to 0.3 log CFU/g. These studies provide a very important insight into the application of bacteriophages in meat: they show that phages are also effective against multidrug-resistant isolates. Currently, the growing bacterial resistance to antibiotics is a huge problem. Therefore, new methods of eliminating such bacterial strains are of great interest. Bacteriophages seem to be a promising solution.

**In summary,** according to the presented literature data, bacteriophages are promising antibacterial agents, but much research is still required to obtain effective phage preparations. The commercial phage preparations available on the market often do not completely eliminate pathogens in meat. For instance, SalmoFresh^TM^ reduced bacterial counts by 0.7–1.3 log units [50], and Phage Guard S by 1.0–2.9 log units [51,54,55]. ListShield was the most effective and reduced bacterial counts by 3.5 log units [72]. According to Commission Regulation (EC) No 2073/2005 of 15 November 2005 on microbiological criteria for foodstuffs, for several meat and meat products, the following criteria must be met: *Salmonella* sp. (absent in 10 g or 25 g) and *Listeria monocytogenes* (absent in 25 g or 100 CFU/g). In experimental conditions, meat is usually inoculated with a relatively high bacteria inoculum of ≥10^3^ CFU/g. Usually, the pathogen concentration in meat treated with bacteriophages does not meet the above criteria. However, under real conditions, the number of pathogens is usually lower, and it is possible to completely inactivate bacteria and meet the mentioned criteria. It is very promising that the effectiveness of phage preparations is often comparable to that of chemical preservatives—sometimes phage preparations have stronger activity [50]. Usually, bacteriophages possess a narrow spectrum of action and are effective only against a single bacterial strain, serotype, or species. However, some studies show that bacterial viruses can also have inhibitory properties against different bacterial genera [57]. Unfortunately, bacteriophages are sensitive to heat treatments, which cause the loss of their lytic properties. However, pathogenic bacteria are also sensitive to high temperatures and should not be viable after the cooking process. Undoubtedly, one of the most important advantages is the antibacterial activity of virions against MDR microorganisms. Slaughter animals, especially poultry, are often fed antibiotics, which is not recommended and do not ensure protection from MDR bacteria.

## 5. Application of Bacteriophages to Control the Growth of Meat Spoilage Bacteria

The knowledge about the application of bacteriophages to inhibit the growth of meat spoilage bacteria is very limited. Studies focus mainly on controlling the growth of pathogenic bacteria, such as *Salmonella* sp., *L. monocytogenes*, *E. coli* O157:H7, and *C. jejuni*, in meat. However, new methods of meat preservation that can serve as alternatives to chemical preservatives are required. Bacteriophages could suppress the growth of bacteria and could therefore inhibit microbiological spoilage processes in meat.

Greer [91] found that bacteriophages can inhibit the growth of *Pseudomonas* sp. in beef meat. A significant reduction in bacterial number from days 1 to 4 of storage was observed in steak. On day 1, a decrease from 4.7 log CFU/cm2 to 3.6 log CFU/cm2 was detected in the phage-treated sample. However, after 2 days, bacterial counts sharply increased, but their levels were still significantly lower than in the control. In addition, the extent of steak surface discoloration in samples containing bacteriophages was significantly lower compared to the control. Greer and Dilts [92] investigated the ability of bacteriophages to control the growth of *Brochothrix thermosphacta* in pork adipose stored at 2 and 6 °C. The authors demonstrated that under both storage conditions, phages reduced the bacterial number by about 2 log units during 2 days of storage compared to the control. Although bacterial recovery was observed, their numbers were still lower than in the control. In addition, the reduction in *B. thermosphacta* counts by bacteriophages decreased odor development. The samples containing phages remained acceptable throughout 8 days of storage at 2 °C, whereas the odor of the control sample was unacceptable after 4 days of storage. In a later study, Greer et al. [93] demonstrated the use of bacteriophages to control the growth of *Leuconosctoc gelidurn* in pork adipose stored at 4 °C in air and under vacuum conditions. Phages were more effective at a high MOI than at a low MOI. Although phage replication was not affected by the storage atmosphere, the inhibitory effect of phages in air was stronger than under vacuum conditions. Under both storage conditions, a reduction of 1–2 log units compared to the control was observed from days 2 to 9 of storage.

Although the literature data concerning the inhibitory effect of bacteriophages on spoilage bacteria in meat are very limited, the results are very promising. They show that phages effectively inhibit bacterial growth in meat, which also delays spoilage processes, extends the shelf life, and increases the acceptance of meat.

Some data demonstrate the application of bacteriophages in other food products in order to suppress the growth of spoilage, non-pathogenic bacteria, but they are also very limited. For instance, Tanaka et al. [94] reported that bacteriophages reduced the number of *Pseudomonas lactis* in skim and whole milk. Bacterial counts decreased by 1000-fold compared to the control and remained low for 5 days. Deasy et al. [95] demonstrated the application of bacteriophages to control the growth of *Lactobacillus brevis*—beer spoilage bacteria. At the lowest inoculation level (10^3^ CFU/mL of *L. brevis*), viable bacterial counts were below the detection limit in phage-treated samples within the first 24 h of the trial. At higher inoculum levels of 10^4^ and 10^5^ CFU/mL, the same effect was observed after 48 h. Moreover, no bacterial regrowth was detected.

**In summary**, more studies should focus on investigating the influence of bacteriophages on food spoilage bacteria. In the future, phage cocktails targeting various species of meat spoilage bacteria could be developed. Such cocktails could be used as natural biological meat preservatives and limit the use of harmful chemical substances. However, the development of such phage cocktails requires substantial research, including studies on interactions between bacteriophages. Such diversity of bacterial viruses can contribute to competition between them and the loss of the preparation’s effectiveness.

## 6. Synergistic Effects of Bacteriophages and Other Antimicrobial Agents

Some studies have focused on investigating the synergistic effects of bacteriophages and antimicrobial agents. Studies show that a mixture of bacteriophages and antimicrobial agents can exert synergistic effects, exerting stronger antibacterial effects than either agent used alone [96]. This seems to be a promising research direction. Bacteriophages are effective against only a narrow group of microorganisms—usually influencing one bacterial species. A mixture of bacteriophages and different antimicrobial agents can broaden the range of activity. Both natural and chemical antimicrobial substances have been investigated. Although chemical preservatives can be harmful to human health, using bacteriophages enables their application at lower concentrations, which may be safer. Such a synergistic preparation can also show antioxidant properties and suppress oxidative changes in meat and meat products, preventing spoilage processes. The literature data also reveal that bacteriophages can be applied together with physical fixation methods, such as UV light and high pressure, yielding synergistic effects.

According to Sukumaran et al. [50], the phage preparation SalmoFresh^TM^, together with the chemical antimicrobials lauric alginate and cetylpyridinium chloride, significantly reduced *S.* Typhimurium, *S.* Enteritidis, and *S.* Heidenberg counts compared to the control, phage preparation alone, and chemical antimicrobials used alone. In broth, the effect was already observed after 2 h of storage at 4 °C; however, after 24 h, the mixtures of bacteriophages (both 10^8^ and 10^9^ PFU/mL) with antibacterial agents decreased *Salmonella* sp. counts, in most cases to undetectable levels. The effect was weaker when applied to chicken breast fillets; during 7 days of storage, the mixtures reduced *Salmonella* sp. levels by 0.9–1.4 log units compared to the control. Nevertheless, SalmoFresh^TM^ applied alone decreased *Salmonella* counts by 0.5–1.1 log units compared to the control.

Moon et al. [97] reported that the commercial bacteriophage preparation PhageGuard S™ showed a synergistic effect with terpenes from essential oils and caused significantly greater reduction in *Salmonella* sp. than when used separately. Thymol alone at 0.8 and 1.6% inactivated *Salmonella* sp. by 0.6 and 1.3 log units, respectively. Bacteriophages decreased bacterial counts by 0.9 log units immediately after treatment. However, a mixture of bacteriophages and thymol (0.8 and 1.6%) resulted in 1.5 and 1.9 log unit reductions in *Salmonella* sp. on chicken meat, respectively. With carvacrol (0.8 and 1.6%), reductions of 0.7 and 1.6 log units were achieved immediately after treatment. When it was combined with bacteriophages, *Salmonella* sp. counts decreased more significantly (by 1.7 and 2.0 log units, respectively).

Wang et al. [98] investigated a combination of bacteriophages, nisin, and potassium sorbate on the shelf life of chilled pork. The results of these studies demonstrated that nisin and potassium sorbate did not affect *Salmonella* sp. counts but decreased TVC in fresh chilled pork. Bacteriophages were active mainly against *Salmonella* sp. However, their combination significantly decreased TVC and completely eliminated *Salmonella* sp. This was the best antimicrobial combination; after 14 days of storage, TVC was about 10^5^ CFU/g, whereas in the control, it was 10^7^ CFU/g. In addition, it prevented color parameter changes, lipid oxidation, and organoleptic changes. These studies suggest that bacteriophages in combination with nisin and potassium sorbate have a synergistic effect and can together reduce the counts of foodborne pathogen *Salmonella* sp., inhibit spoilage processes, and consequently extend the shelf life of meat more effectively.

Dykes and Moorhead [99] used bacteriophages and nisin in vacuum-packaged beef stored at 4 °C for 4 weeks. Phages alone did not significantly influence the number of *L. monocytogenes*. However, the combination of bacteriophages and nisin reduced bacterial counts by about 1 log unit. In the meat sample treated only with nisin, the *L. monocytogenes* load was about 0.5 log units lower, which suggests that bacteriophages and nisin show a synergistic effect.

Chibeu et al. [100] investigated the use of the phage preparation Phage Guard L™ alone or in combination with chemical antimicrobials (potassium lactate and sodium acetate) in cooked turkey and roast beef stored at 4 and 10 °C. Generally, at the beginning, phages used without any additional antimicrobials decreased the number of *L. monocytogenes* compared to the initial state, but later, regrowth was observed. However, phages also showed synergistic effects with chemical antimicrobials. In turkey samples stored at 4 °C, Phage Guard L™ with potassium lactate caused a large reduction of 4 log units. In beef meat stored at 10 °C and treated with phages, sodium acetate, and potassium lactate, the bacterial number was stable (about 1.6–1.8 log CFU/g) for 10 days and increased on day 28, reaching 6.3 log CFU/g. Pelyuntha and Vongkamjan [101] reported a synergistic effect of bacteriophages with propionic acid against *Salmonella* sp. in chicken meat stored at 4 °C in MAP. The combined treatment caused a reduction to below the detection limit (reduced by 4 log units) on day 2 of storage. In samples treated with propionic acid and the phage cocktail separately, a complete reduction in bacteria was observed on day 4 of storage.

Yeh et al. [102] investigated the effects of bacteriophages, chemical antimicrobials, UV light, and their combinations on *Salmonella* sp. in ground beef. The phage cocktail alone diminished bacterial levels by 1.2 units. It was more effective than organic acids and showed a similar influence to that of UV light. When used in combination, bacteriophages significantly enhanced the activity of lactic and peroxyacetic acids, as well as increased the antibacterial effect of UV light. The highest efficiency was achieved in samples treated with a combination of the phage mixture and UV light—a reduction of 2.0 log units was observed. Yang et al. [103] demonstrated the influence of combined treatment with UV-C light and the phage preparation ListShield^TM^ on *L. monocytogenes* in chicken breast meat stored at 4 °C. Generally, phage treatment reduced bacterial counts by a maximum of 0.8 log units when used alone, but in combination with UV-C treatment, a reduction of 2.0 log units during 72 h storage was observed. In addition, no significant differences in thiobarbituric acid reactive substances (TBARS), pH, surface color, or other sensory attributes were observed during storage. Komora et al. [104] investigated the effect of high-pressure processing assisted by the bacteriophage Phage Guard L™ and bacteriocinogenic *Pediococcus acidilactici* on *L. monocytogenes* in fermented meat sausage. The phage preparation alone was unable to eliminate *L. monocytogenes* over the 60 days of storage. However, under high pressure (300 MPa) combined with phages, the *L. monocytogenes* number decreased immediately to below the detection limit, with more than a 3.0 log decline. In the sample treated with *P. acidilactici* and Phage Guard L™, the reduction in bacterial counts was mainly due to *P. acidilactici* because the phage titer decreased over the storage period, probably due to the acidification caused by lactic acid bacteria.

Shebs et al. [105] reported synergistic effects of bacteriophages and peroxyacetic acid, acidified sodium chlorite, and ultraviolet light on *E. coli* O157:H7 in beef trim. Bacteriophages increased the effectiveness of the other treatments.

**In summary**, the literature data show that bacteriophages combined with other antimicrobial agents can have synergistic effects. The combination of these antibacterial agents can reduce the concentrations used. There is also a lower risk of negative organoleptic changes when natural substances such as essential oils or polyphenolic extracts are applied. Using lower doses of chemical preservatives by combining them with phages can minimize their harmful health effects. However, in-depth investigation is needed to determine whether bacteriophages are stable in the presence of these agents and whether their lytic activity is inhibited. The literature data reveal that some of them, e.g., essential oils and bacteriocins, possess antiviral properties [106,107].

## 7. Application of Endolysins to Control Bacterial Growth in Meat

Studies show that bacteriophages can be effective agents to control the growth of pathogenic and spoilage bacteria in meat. However, some studies report the application of endolysins in food. Endolysins or lysins are proteins synthesized by bacteriophages in the late infection stages. They kill bacteria by damaging peptidoglycan in the cell wall. The mass of endolysins that target Gram-positive bacteria tends to be between 25 and 40 kDa. They have two functional domains: a domain responsible for their catalytic activity (EAD—enzymatically active domain) and a domain for substrate recognition (CBD—cell-wall-binding domain). In contrast, endolysins targeting Gram-negative bacteria possess only a single catalytic domain and are smaller in size (15–20 kDa) [10,107,108].

The antibacterial activity of endolysins has been widely investigated in the literature. Compared to bacteriophages, they show a more extensive spectrum of inhibitory activity against the growth of many bacterial strains or species. In addition, bacterial resistance to endolysins has not been reported yet. However, they remain underestimated and have been poorly investigated as natural preservatives of meat [7,12,109].

Yuan et al. [110] reported that the endolysin Abtn-4 obtained from *Acinetobacter baumanii* bacteriophage D2 reduced *A. baumanii* viable counts by more than 3 log units. In addition, it inhibited the growth of some Gram-positive and Gram-negative bacteria: *Staphylococcus aureus*, *Pseudomonas aeruginosa*, *Klebsiella pneumoniae*, *Enterococcus* sp., and *Salmonella* sp. Moreover, the endolysin revealed activity against phage-resistant strains. Kim S. et al. [111] found that the recombinant LysSAP26 endolysin can show inhibitory activity against MDR bacteria: carbapenem-resistant *A. baumannii*, *E. coli*, *K. pneumoniae*, and *P. aeruginosa*, oxacillin-resistant *S. aureus*, and vancomycin-resistant *Enterococcus faecium*. Zhang et al. [112] demonstrated the antimicrobial activity of LySTG2—an endolysin obtained from the phage STG2 directed against *S.* Typhimurium. This enzyme inhibited the growth of the Gram-negative bacteria *Salmonella* sp., *E. coli*, and *Pseudomonas aeruginosa*; however, it was not active against Gram-positive bacteria. According to Jiang et al. [113], LySSP1—an endolysin also obtained from a bacteriophage directed against *S.* Typhimurium—showed antibacterial activity not only against various strains and serotypes of *Salmonella* sp. but also against other species of pathogenic Gram-positive and Gram-negative bacteria*: E. coli*, *Shigella* sp., and *L. monocytogenes*. Li et al. [114] showed that the endolysin LysP152 had broader antibacterial activity against the *Staphylococcus* genus than the bacteriophage STAP152. Chang et al. [115] reported changes in *S. aureus* morphology after exposure to the LysSA97 endolysin. The cells were shriveled. Deformation of the cell wall might be due to the activity of N-acetylmuramoyl-L-alanine amidase, which breaks the bonds between N-acetylmuramoyl residues and L-alanine within the peptidoglycan layers [116].

Some studies have focused on investigating the antibacterial activity of only the lytic domain—not the whole enzyme—but the results are inconsistent. According to Gu et al. [117], the activity of the catalytic domain CHAP_LysGH15_ (cysteine, histidine-dependent amidohydrolase/peptidase) was weaker than that of the native enzyme, LysGH15. In contrast, Horgan et al. [118] demonstrated that the antibacterial effect of the CHAP_LysK_ domain was twice as strong as that of LysK.

Endolysins were also applied in meat to inhibit bacterial growth, but limited literature data were found. Yan et al. [119] compared the influence of LysGH15 and its domain CHAP_LysGH15_ on MRSA *S. aureus* in Chinese bacon and pork. The endolysin showed much stronger activity, and at a concentration of 0.4 nmol/cm^2^, bacteria were completely eliminated after 2 h exposure. At the same concentration, CHAPL_ysGH15_ decreased viable counts by only 0.7 log units. In pork, the effect was the opposite. *S. aureus* was below the detection limit when it was treated with 0.4 nmol/cm^2^ of CHAPLysGH15, but after exposure to endolysin, only a reduction of 1.0 log units was observed. These differences in results for bacon and pork are probably caused by different salt contents. In bacon, the salt content was high (10.5%), whereas in pork, it was very low. The authors also investigated the influence of salt concentration on the activity of LysGH15 and CHAP_LysGH15_ and demonstrated that high NaCl content suppresses the activity of the lytic domain, whereas it increases the activity of the endolysin. Hou et al. [120] confirmed that endolysins show stability across a wide range of NaCl concentrations, which is important for meat processing. The authors also indicated that the endolysin Lys1472 reduced the *Clostridium perfringens* count by about 3 log units during 12 h of storage at 4 °C and 6 h at 25 °C. Li et al. [113] demonstrated that LysP152 had a greater inhibitory effect on MRSA *S. aureus* in pork meat stored at 4 °C than the bacteriophage STAP152. During 24 h of storage, bacterial counts decreased to low levels. Guan et al. [121] indicated that the endolysin LysPFX32 effectively inhibited the growth of *Pseudomonas fluorescens* in pork meat in a concentration-dependent manner. The bacterial counts in control samples inoculated with bacterial suspensions of 10^5^, 10^3^, and 10^1^ CFU/g increased to 9.3, 8.7, and 7.9 log CFU/g, respectively. In meat treated with 95 μg/mL of LysPFX32, they increased to 6.5, 6.1, and 5.8 log CFU/g, respectively.

Studies show that endolysins can have synergistic effects with different antimicrobials. Kim et al. [122] reported that essential oils and LysPB32 inhibited *S.* Typhimurium more significantly than when used individually. Their combinations reduced the *S*. Typhimurium count to below the detection limit after 12 h incubation at 37 °C. Chang et al. [114] investigated the synergistic effect of the LySA97 endolysin and carvacrol in different food matrices—skin, whole milk, and lean beef—against *S. aureus*. In meat, a tenfold higher enzyme concentration was needed, probably due to the complexity of the meat matrix. The endolysin alone showed only a minor antibacterial effect. However, when applied with carvacrol, it significantly increased the activity of this compound, indicating a synergistic effect. After 3 h, a reduction of 2.1 log units was achieved.

**In summary,** the application of endolysins in meat is innovative and can be beneficial in the future. Endolysins can be effective against MDR microorganisms, do not cause bacterial resistance, and have a broader range of activity than bacterial viruses. They may become an alternative to bacteriophages in the future when bacterial resistance to bacteriophages increases. However, the current procedure for endolysin isolation is complicated, and purchasing them is expensive. Therefore, they are not commonly applied in food, including meat.

## 8. Advantages and Disadvantages

**Figure 4:**
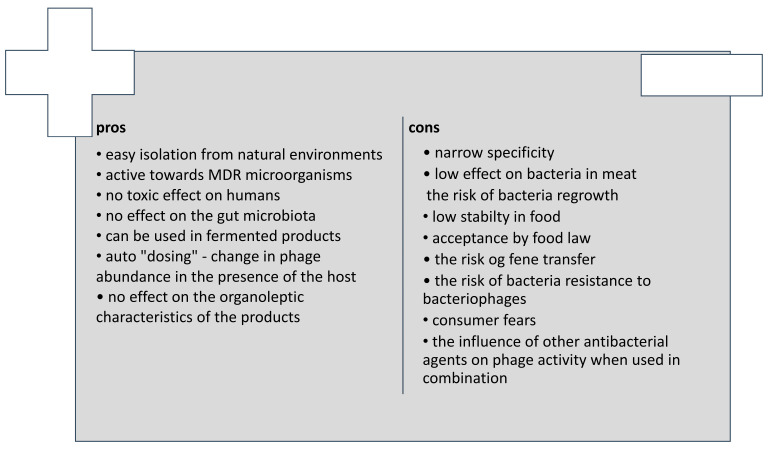
Advantages and disadvantages of the application of bacteriophages in meat [7,8,14].

The application of bacteriophages in meat has some advantages and disadvantages (Figure 4). Bacteriophages are generally ubiquitous in the natural environment and considered safe to humans. The procedure for their isolation from the natural environment and subsequent propagation is easy and not cost-intensive. They exhibit antibacterial properties against MDR microorganisms and are capable of multiplying in the presence of host bacteria. Consequently, their levels should not diminish during food storage. Contrary to antibiotics, they do not influence human commensal microbiota; they possess a narrow range of activity and inhibit the growth of target bacterial species/strains. In addition, due to their narrow specificity, they can be used in meat products obtained with microorganisms, e.g., lactic acid bacteria, without the risk of affecting the technological process. Finally, they do not cause organoleptic changes in food and are generally stable in meat during storage.

However, there are also some limitations to using bacteriophages in food. On the one hand, the narrow host spectrum of bacteriophages is an advantage, as mentioned above. On the other hand, this necessitates searching for bacteriophages with a broad spectrum of action or designing phage mixtures to increase the efficacy of phage preparations applied in food. As far as phage cocktails are concerned, the diversity of bacterial viruses in one preparation can lead to competition between phages, which can lower antibacterial activity. Therefore, the available commercial phage preparations target a specific bacterium. The development of phage preparation targeting a broad spectrum of bacterial species is very complicated and requires considerable research [7,8,14].

Another drawback is the relatively weak lytic activity of bacteriophages in the meat matrix. Interestingly, those available on the market reduce bacterial counts only by 1–2 log units. This weak influence on bacteria in meat is a serious concern. Phage preparations are mainly applied to the meat surface, which makes it difficult for them to contact bacteria located deeper in the matrix. The complicated microstructures and matrices of foods affect the lytic properties of bacteriophages by decreasing phage diffusion or interfering with virions, consequently worsening their ability to contact bacteria. The lytic activity of bacteriophages in meat depends mainly on the inoculum level, phage concentration, and temperature of storage. The higher the MOI, the stronger the activity of the bacteriophage. Thus, a high phage titer should be applied in meat to increase the likelihood of individual bacteria contacting at least one phage. It should be emphasized that in experiments, relatively high bacterial concentrations are used as inocula. In most real-life cases of food contamination, pathogen levels are low, so it is then possible to completely inactivate bacteria. Some literature data indicates a risk of bacterial regrowth in food during long storage periods. Although bacteriophages replicate in food matrices, there are some doubts about whether they prevent recontamination. In addition, bacteriophages can be unstable when applied to food because many factors, such as temperature, pH, and salt concentration, can influence protein stability during food processing. Some ingredients present in food can also contribute to their reduced antibacterial activity. Moreover, there is a risk of mutants occurring in food that are unsusceptible to bacteriophages [7,8,14]. Although bacterial resistance to bacteriophages is not common yet, it is possible and has been described in the literature. Bacteria can become resistant to phages through spontaneous mutations in genes associated with the biosynthesis of phage receptors, which leads to decreased attachment and virulence [123]. Finally, the literature data indicate that bacteriophages show synergistic effects when combined with other antibacterial agents. However, these factors can decrease their lytic properties and their ability to infect bacterial cells [96].

These limitations contribute to legal barriers in the EU to the acceptance of bacteriophages’ application in food. EFSA has not yet given a positive opinion to any proposed phage preparations [32,33,34]. In addition, the fact that bacteriophages are viruses can cause fear in consumers.

## 9. Summary

In recent years, due to the increasing number of MDR microorganisms, bacteriophages have garnered significant interest in both medicine and the food industry. Studies show that they can be applied to food, including meat, and prevent bacterial growth. Most studies have focused on investigating the use of bacteriophages as biocontrol agents to prevent the growth of foodborne pathogens. The application of phages to inhibit the growth of spoilage microorganisms in meat has been poorly described. This seems to be an interesting research direction to develop phage preparations consisting of a wide range of virions that can inhibit microbial spoilage processes. This requires considerable research, including studies on interactions between phages in phage cocktails. Some investigations have focused on the synergistic effects of bacteriophages and other antibacterial agents. The mixture of bacteriophages and antimicrobial substances, both chemical and natural, enables their concentrations to be reduced, minimizing the risk of organoleptic changes and/or harmful effects to human health, as well as extending the spectrum of action. Such combinations can not only effectively inhibit the growth of pathogenic and spoilage microorganisms but also suppress physicochemical changes, such as oxidative processes and color changes. Finally, more studies must be performed on the application of endolysins synthesized by bacteriophages—they possess a wider range of antimicrobial activity than phages, and currently, bacterial resistance to these toxins is unknown.

Although there are some limitations to using bacterial viruses in food, bacteriophages have great potential to be used as antibacterial agents in meat. Further research is needed to design safe and effective phage preparations and obtain a positive EFSA opinion. The intensity of phage research indicates that bacteriophages can be commonly used in the food sector, just as they are now used in medicine.

## Figures and Tables

**Table 1:** Commercial phage preparations dedicated for application in food [35].

Target Microorganisms	Phage Preparation	Producer	Food Products Dedicated	Certification
*Salmonella enterica (*serotypes *Typhimurium*, *Enteritidis*, *Heidelberg*, *Newport*, *Hadar*, *Kentucky*, *Thompson*, *Georgia*, *Agona*, *Grampian*, *Senftenberg*, *Alachua*, *Infantis*, *Reading, and Schwarzengrund)*	SalmoFresh™	Intralytix (Columbia, MD, USA)	poultry, red meat, fish and shellfish, and fresh and processed fruits and vegetables	Kosher; Halal; OMRI
			raw and ready-to-eat poultry products, raw and ready-to-eat red meat products	
			fish, shellfish, fresh and processed fruits and vegetables and poultry immediately before or after grinding, and on ready-to-eat products before slicing	
			fish, shellfish, and fresh and processed fruits and vegetables, or on ready-to-eat poultry products prior to slicing and on raw poultry prior to grinding or after grinding	
*Salmonella* sp.	PhageGuard S™ (previously Salmonelex^TM^)	Phageguard Micreos Food Safety (Wageningen, The Netherlands)	Meat and poultry	Kosher; Halal; OMRI; SKAL
*Salmonella* sp.	SalmoPro^®^	Phagelux	poultry, red meat, fruits, vegetables, eggs, fish, and shellfish	
*Listeria monocytogenes*	ListShield™	Intralytix (Columbia, MD, USA)	ready-to-eat meat and poultry products, smoked salmon, fresh and processed fruits and vegetables, dairy products (including cheese)	Kosher; Halal; OMRI
*Listeria monocytogenes*	Listex™ (Phage Guard L^TM^)	Phageguard Micreos Food Safety (Wageningen, The Netherlands)	ready-to-eat meat and poultry products, fresh salmon, fresh scallops and shrimp	Kosher; Halal; OMRI; SKAL
*Escherichia coli* STEC including *E. coli* O157:H7	EcoShield PX™	Intralytix (Columbia, MD, USA)	meat, poultry, fruits, vegetables, dairy products (including cheese), fish, and other seafood	Kosher and Halal
*E. coli* O157:H7	PhageGuard E^TM^	Phageguard Micreos Food Safety (Wageningen, The Netherlands)	Beef carcass and parts, leafy green vegetables	-
*Shigella sp.* (*S. flexneri*, *S. sonnei* and *S. dysenteriae*)	ShigaShield^TM^	Intralytix (Columbia, MD, USA)	ready-to-eat meat and poultry, fish (including smoked fish), shellfish, fresh and processed fruits and vegetables, and dairy products including cheese	-

## Data Availability

No new data were created or analyzed in this study. Data sharing is not applicable to this article.

## Supplementary Materials

The following supporting information can be downloaded at https://www.mdpi.com/article/10.3390/molecules30173641/s1, Table S1: Application of bacteriophages in meat.
