# Supplementary material for: Bacteriophage Power: Next-Gen Biocontrol Strategies for Safer Meat
Source: Molecules. 2025 Sep 6;30(17):3641. doi: 10.3390/molecules30173641 (PMC12430668; doi:10.3390/molecules30173641)
Supplement: Supplementary file 1 [file molecules-30-03641-s001.zip › molecules-3805898-supplementary.pdf]

Table S1. Application of bacteriophages in meat

Meat samples	Target bacteria	Bacteria inoculation level	Commercial phage preparation	Bacteriophages concentration or MOI	Storage temperature	Time of storage	Maximum reduction of bacteria counts (compared to control)	Additives used	Refere nce
Cooked meat products (turkey ham and chicken sausage), cured sausages (Italian salami and barbecue sausage)	*Salmonella* Enteritidis	10^3CFU/mL; 10^4 CFU/mL; 10^5 CFU/mL	-	10^8 PFU/mL<br>10^9 PFU/mL	4°C<br>18°C	10 days	By 2 log units	-	[45]
Chicken meat	*Salmonella* Typhimurium	10^4 CFU/mL		10^8 PFU/ml<br>10^7 PFU/ml<br>10^6 PFU/ml	4°C 7<br>25°C	7 days<br>7 hours	By 2.5 log units	-	[43]
Chicken meat	*Salmonella* Typhimurium	10^4 CFU/g		10^9 PFU/mL	10°C<br>20°C<br>30°C	72 hours	By 1.4 log units	-	[44]
Chicken meat	*Salmonella* Enteritidis and *Salmonella* Typhimurium	3×10^4 CFU/meat piece (4 cm^2)	-	3×10^8 PFU/meat piece (4cm^2)	8°C<br>25°C	24 h	Below a detection limit (by 3.1 log units)	-	[16]
Chicken meat	*Salmonella* Typhimurium and *Salmonella* Enteritidis	10^6 CFU/mL	-	10^9 PFU/mL	4°C	7 days	By 2.2 log units (compared to initial state)	-	[48]
Beef and chicken meat	*Salmonella* Enteritidis	10^5 CFU/mL	-	10^{12} PFU/mL	4°C	48h	By 2.2 log units	-	[23]
Chicken breast meat	*Salmonella* Enteritidis	9.5 × 10^5 CFU/mL	-	MOI=10^4 PFU/CFU<br>MOI=10^5 PFU/CFU<br>MOI=10^6 PFU/CFU	4°C	7 days	By about 4 log units	-	[17]
Chicken meat and chicken skin	*Salmonella* Enteritidis	10^3 and 10^6 CFU/g	-	10^9 PFU/g	4°C<br>25°C	8 days<br>4 days	Below the detection limit (by about 6,4 log units)	-	[22]
Turkey meat	*Salmonella enterica* subs. enterica	10^7CFU/mL	-	10^8PFU/mL	4°C	6h	By 1.4 log units	-	[53]

Bbeef, pork, chicken and turkey meat	*Salmonella enterica*, *Salmonella* Heidelberg, *Salmonella* Newport	10^7CFU/g	-	10^7 PFU/mL<br>10^8 PFU/mL	4°C	6h (chicken and turkey)<br>18 h (beef and pork)	By 1.1 log units	-	[55]
Chicken breast meat	*Salmonella* Typhimuriu	10^8 CFU/mL	-	10^8 PFU/mL	25°C	6h	By 2.2 log CFU/g		[56]
Chicken meat	*Salmonella* Typhimurium, *Salmonella* Heidelberg, and *Salmonella* Enteritidis	10^3 CFU/g	SalmoFresh™	10^9 PFU/mL	4°C	7 days	By 1.2 log units	-	[50]
Turkey breast cutlets, ground turkey	*Salmonella* Heidenberg	10^3 CFU/g	SalmoFresh™	10^7 PFU/g	4°C	24h	By 1.3 log units	-	[49]
Chicken meat	*Salmonella* Typhimurium, *Salmonella* Newport, *Salmonella* Thompson, *Salmonella* Heidelberg, and *Salmonella* Enteritidis	10^4 CFU/g	PhageGuard S™	10^7 PFU/cm^2	4°C	8h	By 0.9 log units	-	[51]
Beef meat	*Salmonella sp.*	10^4 CFU/g	PhageGuardS ™	10^8 PFU/g<br>10^9 PFU/g	5°C	30 min	By 1.7 log units	-	[54]
Duck meat	*Salmonella* Typhimurium	3.3 × 10^4 CFU/cm^2<br><br>9.3 × 10^2 CFU/cm^2	-	MOI=10^4 PFU/CFU<br>MOI=10^5 PFU/CFU<br><br>MOI=10^4 PFU/CFU<br>MOI=10^6 PFU/CFU	4°C<br>25°C	7 days<br>12h	Below a detection limit (by 5.8 log units)	-	[52]
Chicken meat and chicken skin	*Salmonella* Typhimurium, Salmonella Heidenberg and *Salmonella* Enteritidis	10^3CFU/g	SalmoFresh™	10^9 PFU/mL<br>10^8 PFU/mL	4°C	7 days	By 1.4 log units	Lauric arginate and cetylpyridinium chloride	[30]

Pork meat	*Salmonella* Typhimurium	10^3 CFU/g	-	10^9 PFU/g	4°C	21 days	By about 2.5 log units	Nisin and potassium sorbate	[98]
Chicken meat	*Salmonella* Typhimurium and *Salmonella* Enteritidis	10^5CFU/mL	PhageGuard S™	10^8 PFU/mL<br>10^9 PFU/mL	4°C	5 days	By 2 log units	Thymol and carvacrol	[97]
Beef meat	*Salmonella* Infantis, *Salmonella* Heidelberg, *Salmonella* Newport, streptomycin resistant *S.* Enteritidis C	3.5 log CFU/g	PhageGuard S™	10^8 PFU/mL	5°C	1h and 30min	By 2 log units	Lactic acid, peroxyaceti c acid, UV ight	[102]
Chicken meat	*Salmonella* Enteritidis, *Salmonella* Hadar, *Salmonella* Infantis, *Salmonella* Typhimurium, *Salmonella* Virchow	10^4 CFU/mL	-	10^7 PFU/mL	4°C	5 days	By 3.9 log units	Propionic acid	[101]
Ready to eat duck meat	*Salmonella enterica*	10^4 CFU/g	-	10^8 PFU/g	4°C	2 days	By 1.8 log units		[18]
	Escherichia coli O157:H7						By about 2 log units		
Mutton and chicken meat	*Campylobacter jejuni*	10^4 CFU/mL (20g)	-	10^6 PFU/mL	4°C	48h	By 1.5 log units	-	[24]
Chicken skin	*Campylobacter jejuni*	10^4 CFU/mL ($12cm^2$)	-	10^7 PFU/mL ($12cm^2$) of each phage	5°C	24h	By 0.73 log units	-	[62]
Chicken meat	*Campylobacter jejuni* *Campylobacter coli*	-	-	MOI=10^2 PFU/CFU	4°C	168h	No influence	-	[63]
Chiken liver	*Campylobacter jejuni*	10^3 and 10^5cfu/mL	-	10^8 PFU/g	4°C	48h	By 0.7 log units		[64]
Chicken skin	*Campylobacter jejuni*	10^4 CFU/g	-	MOI = 10^4 PFU/CFU	4°C	48h	By about 4 log units (to non detectable level)		[65]

Sirloin steaks	*Listeria monocytogenes*	10^9 CFU/g (befoe thermal processing) 10^3-10^4 CFU/g (after thermal processing)	-	10^9 PFU/g	4°C	28 days	Below a detection limit	-	[73]
Cooked turkey and roast beef	*Listeria monocytogenes*	10^3 CFU/cm^2	PhageGuard L™	10^7 PFU/cm^2	4 and 10°C	28days	By about 6.4 log units	Potassium lactate and sodium acetate	[100]
Vacuum packed beef meat	*Listeria monocytogenes*	-	-	3×10^3 PFU/mL	4°C	28 days	By about 1.5 log units	nisin	[99]
Chicken breast meat	*Listeria monocytogenes*	4.5 log CFU/g	ListShield™	5×10^8 PFU/g	4°C	72 hours	By about 2 log units	UV-C irradiation at different doses of 600, 1200, 1800, and 2400 mWs/cm^2	[103]
Fermented meat sausage	*Listeria monocytogenes*	-	PhageGuard L™	MOI = 10^3 PFU/CFU	4°C	60 days	Below a detection limit (by 3 log units)	-	[104]
Spanish dry cured ham	*Listeria monocytogenes*	10^5 CFU/cm^2 10^4 CFU/cm^2 10^3 CFU/cm^2	ListShield™ and PhageGuard L™	10^7 PFU/cm^2 (ListShield™) 10^9 PFU/cm^2 (Listex™ P100)	4°C 12°C	14 days	Below a detection limit (by over 7 log units)	-	[72]
Beef meat	*Listeria monocytogenes*	-	ListShield™	1×10^9PFU/ml	4°C	15 days	By 5.8 log units	-	[21]
Beef meat	*Listeria monocytogenes*	4.5 log/cm^2	-	MOI = 10^2, 10^3, 10^4 PFU/CFU	25°C	24h	By 2.9 log units	-	[74]
Beef meat	*Escherichia coli* O157:H7	3 × 103 CFU/g	EcoShield™	3×10^6 PFU/g	4°C	7 days	By about 2 log units		[46]
Beef meat	*Escherichia coli* (STEC) O157:H7	1×10^7 CFU/ml	PhageGuard E™	1×10^{10} PFU/mL	-	30 min 6h	By 1.4 log units	-	[77]
Beef meat	*Escherichia coli* (STEC) O157:H7 and ESBL *Escherichia coli*	3×10^5 CFU/piece	-	3×10^7 PFU/piece	8°C 25°C	24h	By 2.5 log units	-	[80]
Beef meat	*Eschrichia coli STEC strains*	10^5 CFU/g	-	10^8 PFU/mL	25°C 7°C	6h	By 1.3 log units	-	[82]

	(O26, O45, O103, O111, O121, O145, and O157:H7)								
Beef meat	*Escherichia coli O157*	1 × 105 CFU/cm2	-	1 × 10^8 PFU/cm^2<br>2 × 10^7 PFU/cm^2	4°C	54h	By 1.3 log units		[83]
Beef meat	*Escherichia coli* O157:H7	10^3–10^4 CFU	-	10^8 - 10^9 PFU	4°C<br>24°C<br>37°C	48h<br>24h<br>24h	By 4.0 log units	-	[84]
Beef meat	*Escherichia coli* O157:H7	10^4CFU/piece	-	4.4×10^3 PFU/piece<br>4.7×10^5 PFU/piece<br>7.4×10^6 PFU/piece<br>3.2×10^7 PFU/piece	37°C	4h	By over 4 log units	-	[81]
Beef, pork and chicken meat	*Escherichia coli* O157:H7	1×10^5 CFU/cm^2	-	MOI = 10^3 PFU/CFU<br>MOI=10^4 PFU/CFU<br>MOI=10^5 PFU/CFU	4°C<br>37°C	168h	By about 5.5 log units	-	[78]
Chicken meat	ESBL-producing *Escherichia coli*	3×10^4 CFU/piece	-	3×10^7 PFU/piece	25°C<br>5°C	24h	Below a detection limit	-	[79]
Pork meat	*Yersinia enterocolitica*	-	-	MOI=10^4 PFU/CFU	4°C	168h	By about 2 log units	-	[63]
Raw pork meat and ready-to-eat pork	*Yersinia enterocolitica*	10^3 CFU/g	-	1.8 × 10^8 PFU/g	4°C (raw pork)<br>26°C (ready-to-eat pork)	72h (raw pork)<br>12h (ready-to-eat pork)	By about 6.7 log units	-	[20]
corned beef deli meat and pre-cooked chicken	*Shigella sp.*	2×10^3 CFU/g	ShigaShield^{TM}	9×10^5 PFU/g<br>9×10^6 PFU/g<br>9×10^7 PFU/g		48h	By 1.6 log units	-	[87]
Raw and cooked chicken breast	*Shigella flexneri*	10^4 CFU/g	-	10^8 PFU/g	4°C	144h	By about 2 log units	-	[88]
Cooked chicken meat	*Shigella flexneri*	10^4 CFU/g	-	MOI = 10^4 PFU/CFU	4°C	7 days	By about 4 log units	-	[89]
Chicken meat	Multidrug resistant *Shigella flexneri* and *Shigella sonnei*	10^4 CFU/g	-	MOI = 10^4 PFU/CFU	4°C	5 days	By 3.9 log units	-	[90]
Pork adipose	*Brochothrix thermosphacta*	10^6 CFU/cm^2	-	10^3 PFU/cm^2<br>10^5 PFU/cm^2	2°C	10 days	By about 2 log units	-	[92]

		10^5 CFU/cm^2	-	10^5 PFU/cm^2<br>10^6 PFU/cm^2	6°C				
Pork adipose	*Leuconostoc geridurn*	-	-	MOI=10^{-3} PFU/CFU<br>MOI=10^3 PFU/CFU	4°C	12 days	By about 2 log units	-	[93]
Beef steak	*Pseudomonas sp.*	-	-	10^8 PFU/mL	7°C	4 days	By about 2.5 log units	-	[91]
